# Effects of carotenoids on mitochondrial dysfunction

**DOI:** 10.1042/BST20230193

**Published:** 2024-02-22

**Authors:** Opeyemi Stella Ademowo, Olubukola Oyebode, Roshita Edward, Myra E. Conway, Helen R. Griffiths, Irundika H.K. Dias

**Affiliations:** 1 Biomedical and Clinical Science Research, School of Sciences, University of Derby, Derby U.K.; 2 Faculty of Medicine, Health and Life Sciences, Swansea University, Swansea, U.K.; 3 Aston Medical School, College of Health and Life Sciences, Aston University, Birmingham U.K.

**Keywords:** astaxanthin, carotenoids, mitochondria, oxidative stress, reactive oxygen species

## Abstract

Oxidative stress, an imbalance between pro-oxidant and antioxidant status, favouring the pro-oxidant state is a result of increased production of reactive oxygen species (ROS) or inadequate antioxidant protection. ROS are produced through several mechanisms in cells including during mitochondrial oxidative phosphorylation. Increased mitochondrial-derived ROS are associated with mitochondrial dysfunction, an early event in age-related diseases such as Alzheimer's diseases (ADs) and in metabolic disorders including diabetes. AD post-mortem investigations of affected brain regions have shown the accumulation of oxidative damage to macromolecules, and oxidative stress has been considered an important contributor to disease pathology. An increase in oxidative stress, which leads to increased levels of superoxide, hydrogen peroxide and other ROS in a potentially vicious cycle is both causative and a consequence of mitochondrial dysfunction. Mitochondrial dysfunction may be ameliorated by molecules with antioxidant capacities that accumulate in mitochondria such as carotenoids. However, the role of carotenoids in mitigating mitochondrial dysfunction is not fully understood. A better understanding of the role of antioxidants in mitochondrial function is a promising lead towards the development of novel and effective treatment strategies for age-related diseases. This review evaluates and summarises some of the latest developments and insights into the effects of carotenoids on mitochondrial dysfunction with a focus on the antioxidant properties of carotenoids. The mitochondria-protective role of carotenoids may be key in therapeutic strategies and targeting the mitochondria ROS is emerging in drug development for age-related diseases.

## Introduction

The mitochondrion, an important cell organelle, houses the tricarboxylic acid cycle and the electron transport chain (ETC), and plays a crucial role in the production of adenosine triphosphate (ATP) for cell energy, production of reactive oxygen species (ROS), maintenance of calcium homeostasis, initiation of apoptosis and serve as a major hub for cellular iron utilisation [1]. Mitochondria are the main cellular sites of ROS production as well as the primary targets of ROS. ROS, formed by the particle reduction in molecular oxygen including hydrogen peroxide (H_2_O_2_), superoxide anion radicals (O_2_^·−^), and hydroxyl radicals (•OH), are produced as part of normal aerobic life, as by-products of cellular metabolism and could also be produced by exposure to environmental toxins, such as chemical stressors and ultraviolet radiation [2]. ROS have a role in normal physiological function and in redox signalling via different post-translational modifications [2]. At physiological concentrations (nanomolar range), H_2_O_2_ is a signalling molecule involved in stress responses, metabolic regulation, cellular adaptation, proliferation, differentiation, immune defence, and apoptosis [1,2]. Other reactive species similar to ROS and involved in redox signalling include nitric oxide, hydrogen sulfide and oxidised lipids [3]. Cells have different detoxifying and antioxidant mechanisms in place to combat excessive ROS thereby neutralising their damaging effects.

Antioxidants are free radical scavengers that delay or inhibit cellular damage. Natural phytochemicals such as carotenoids have antioxidant properties; they may also induce autophagy, thereby contributing to their antioxidant function [4]. Uncontrolled production of ROS in the absence of adequate antioxidant defence leads to oxidative stress and the consequent molecular damage, contributes to the pathogenesis of several diseases [2,3]. Oxidative stress, a term first used by Sies [5], is described as the imbalance between antioxidants and oxidants in favour of the latter. During oxidative stress, antioxidant levels are low, and the dynamic redox system is disrupted resulting in the higher steady state concentrations of free radicals. The function and dysfunction of mitochondria have been linked to the level of ROS and implicated in age-related diseases, with mitochondrial dysfunction occurring before clinical symptoms [6].

There are four stages in a mitochondrion's life cycle including: biogenesis (co-ordinated expression of mitochondria and nuclear genes by transcription and translation, import of the products and turnover); fusion/fission (critical processes that regulate the size, number, and function of mitochondria due to energy demand) and degradation (removal of dysfunctional mitochondria through the autophagic process, known as ‘mitophagy’). Mitochondrial-targeted antioxidants may promote mitochondrial fusion, decreasing the expression of fission proteins Drp1 and Fis1, thereby decreasing mitochondrial fission [7]. Enhancing antioxidant capacity improves mitochondrial function by ameliorating oxidative stress, inhibiting mitochondrial fission, increasing ATP production, and improving insulin sensitivity. However, many early antioxidant intervention studies were ineffective, and in one major study, antioxidant intervention was associated with negative outcomes [8]. This highlighted the importance of understanding the role of ROS in homeostasis, and the pivot point where oxidative stress impairs normal function.

## Mitochondria and redox homeostasis

Mitochondrial ROS (mROS) are important for major physiological functions, such as signal transduction, cell differentiation and proliferation, wound healing, hypoxic adaptation, and insulin signalling [9]. mROS are produced by complexes I and III of the ETC during ATP synthesis but can affect all other complexes when overproduced, generating additional ROS, which results in mitochondrial dysfunction. mROS can also activate several metabolic sensors including AMP-activated kinase (AMPK), a mitochondrial mechano-redox sensor, and sirtuins which are histone deacetylases [10]. These serve as upstream regulators for the transcription of antioxidant genes, PTEN-induced kinase 1 activity-mediated mitochondrial fission, mitophagy and myosin dependent cell adhesion [10,11]. The metabolic activity of AMPK is also associated with the phosphorylation of the nuclear factor erythroid 2-related factor 2 (Nrf2) and its downstream signalling pathways [12].

Different antioxidant mechanisms allow cells to adapt to environmental stresses for the maintenance of redox homeostasis. These include redox-dependent transcriptional regulatory pathways and endogenous antioxidant system. Glutathione (GSH) is the most abundant endogenous antioxidant molecule, capable of removing ROS directly or indirectly and/or by serving as substrate for different antioxidant enzymes [3]. The indirect mitochondrial protection offered by Nrf2 activation in regulating mitochondrial function and turnover independent of mitochondrial antioxidant and detoxification genes has been reported by several groups [10,12,13]. The interplay between mitochondria and redox homeostasis is a complex and intricately regulated process that impacts various aspects of cellular health. Disruption in mitochondrial redox homeostasis can lead to oxidative stress, mitochondrial dysfunction, and disease development.

## Mitochondrial dysfunction and disease

Mitochondrial function is key to organs with high energy demand and mitochondrial dysfunction contributes to neurodegenerative and cardiovascular diseases [14]. Overall cell health is dependent on mitochondrial quality control (MQC) [15]. Dysfunctional mitochondria may initiate innate immunity through the overproduction of mROS and/or the release of mitochondria DNA; cell death may also be triggered through the release of apoptogenic factors and cytochrome c [1]. MQC involves processes such as proteostasis, biogenesis, mitophagy, mitochondrial dynamics and apoptosis; these ensure cell homeostasis [15]. The maintenance of the MQC processes is important in preventing the development of diseases while ensuring optimal cell function [14,15]. These processes maintain metabolic homeostasis in normal physiology and during cellular stress. Under physiological conditions, mitochondria undergo consistent fission and fusion to ensure homeostasis and energy adaptation but these are disrupted during pathological conditions [7].

The accumulation of dysfunctional mitochondria is associated with the overproduction of mROS, mitochondria-controlled apoptosis, and chronic innate immune activation involved in age related diseases [6]. Mitophagy, which is the process of removing damaged mitochondria is impaired during pathological conditions [7]. Deregulation of MQC processes and ultimately mitochondrial dysfunction has been implicated in sarcopenia [16], glaucoma [17], lung disease [18], infection, obesity, type 2 diabetes/diabetes kidney disease [19], hepatic ischemia-reperfusion injury [20], cardiovascular disease [21], neurological diseases [22] and age-related diseases [9,23].

As ROS have been implicated in pathological conditions, strategies targeting ROS such as lifestyle interventions and dietary supplements are a potential therapeutic focus [7,9]. Healthy lifestyle choices including exercise, diet (eating a diet rich in fibre, fruits and vegetables, unprocessed foods, avoiding too much meat and fat) and dietary supplements have been reported to play a role in MQC, ameliorating mitochondrial dysfunction [24]. Physical activity regulates MQC by allowing the elimination and repair of damaged mitochondria and also promote the synthesis of new mitochondria, recovering the metabolic state [15]. Exercise promotes mitochondrial protein synthesis and has been suggested to protect against Alzheimer's disease (AD) progression by promoting mitochondrial function and cell survival [16].

Poor diet has taken over as a leading cause of death over smoking [24] and many studies have highlighted the importance of diet in health and longevity [24]. Mitochondrial health improves with an antioxidant-rich diet, such as carotenoids, low fat diet, consumption of fibre, plant-based protein, diet rich in polyunsaturated fatty acids and calorie restriction [7,24]. However, mechanisms by which the different antioxidant rich diet or dietary supplements improve mitochondrial function need to be elucidated to eliminate the risk for negative outcomes that was observed previously.

## Carotenoids — free radical scavengers and their protective effects

With exceptions to few water-soluble compounds such as crocin, carotenoids are lipophilic isoprenoid plant pigments with potent anti-inflammatory and antioxidant properties [7]. Carotenoids are synthesised by photosynthetic organisms and some prokaryotes and fungi; animals (including humans) do not produce carotenoids but obtain them in their diets [25]. Most important sources of carotenoids are in fruits and vegetables. However, there are ∼50 carotenoids in daily diets, including from egg yolk and dairy products; it is also important to note that seafoods may supply certain carotenoids (such as lutein, zeaxanthin, astaxanthin, canthaxanthin and fucoxanthin) as most fish and shellfish are rich in complex carotenoids [25].

The absorption, uptake, and transport of carotenoids in mammals involve several complex processes. During digestion, carotenoids are released from the plant matrix and incorporated into mixed micelles in the presence of bile salts. The micelles facilitate the transport of carotenoids across the lipid-rich environment of the intestinal mucosa. Carotenoids are absorbed via intestine lining and incorporated into chylomicrons. Chylomicrons containing carotenoids, along with other lipids, are released into the lymphatic system and then into the bloodstream. In the blood, carotenoids are transported by lipoproteins, with a preference for low-density lipoproteins (LDL), high-density lipoproteins (HDL) and transported to various tissues and organs via the bloodstream [26].

**Figure 1.**
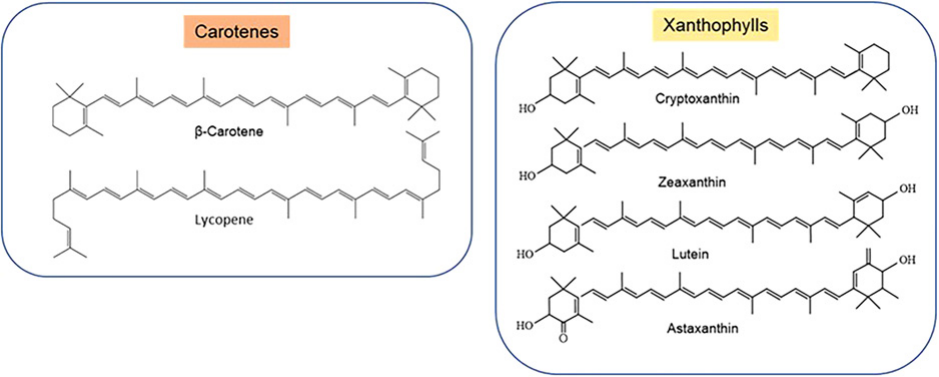
Chemical structures of common carotenes and xanthophylls.

The information including the chemical structures of 1158 carotenoids found in 691 organisms are provided in the Carotenoid Database (http://carotenoiddb.jp), including carotenes (containing carbon and hydrogen atoms only e.g. α-carotenes, β-carotenes, lycopene) and xanthophylls (oxygenated carotenoids with the hydroxyl or epoxide group, e.g. cryptoxanthin, astaxanthin, lutein, zeaxanthin etc.) Carotenes and xanthophylls follow different metabolic pathways are involved in different cellular functions [25] (Figure 1). Carotenoids have been investigated for health benefits in >190 studies, mainly in eye and heart disease [25,27]. Carotenoids protect cells, tissues and organs from the damaging action of singlet oxygen by scavenging free radical via transferring electrons, creating adducts, and moving hydrogen atoms. They protect cells from lipid peroxides and oxygen radicals, play roles in cellular metabolism and function, crop biotechnology and health [25]. Enzymatic cleavage of carotenoid produces biologically active molecules in plants and animals such as hormones and retinoids.

## Oxidative stress and carotenoid metabolism

**Figure 2.**
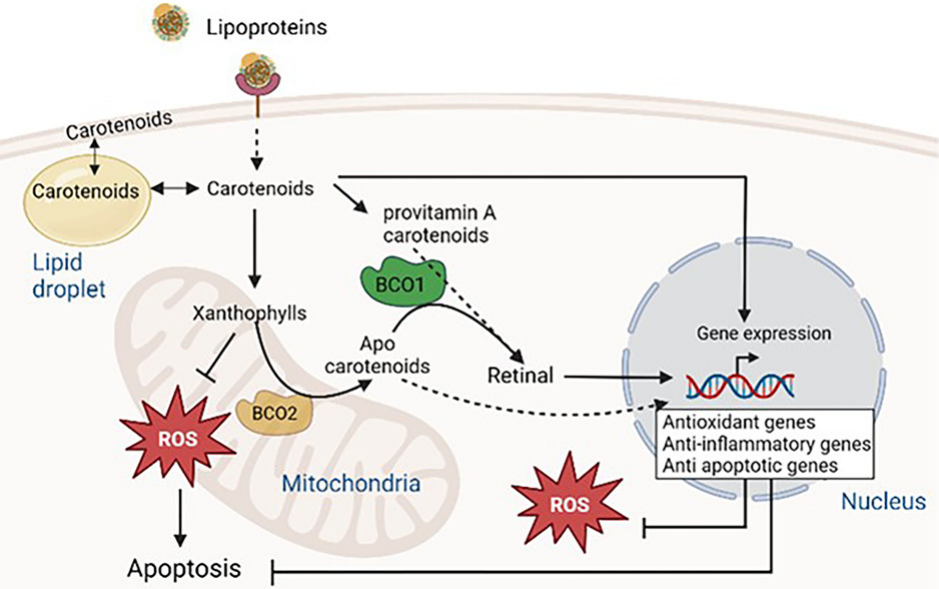
Overview of carotenoid metabolism with relevance to BCO1 and BCO2 enzymes. Carotenoids, found in circulating lipoproteins enter cells through distinct mechanisms involving various receptors and transporters, such as scavenger receptor class B type I, low-density lipoprotein receptor, CD36 membrane transporters, and lipoprotein lipase. Once inside the cells, carotenoids bind to carotenoid-binding proteins and become integrated into cytoplasmic lipid droplets, as well as plasma and mitochondrial membranes. Provitamin A type carotenoids such as β-carotene are symmetrically cleaved by β-carotene oxygenase 1 (BCO1) to yield retinal directly. Mitochondrial BCO2, on the other hand, asymmetrically cleaves carotenoids. The resulting carotenoid cleavage products (apo carotenoids) can be converted into retinal by BCO1. Carotenoids and retinal are transported to the cell nucleus and exert their influence on gene expression (antioxidant genes such as Nrf2 and ERK, anti-apoptotic genes such as Bcl-2 and anti-inflammatory genes) by interacting with nuclear receptors and transcription factors.

Further evidence for the importance of mitochondrial targeted carotenoids comes from research on the human inner mitochondrial membrane enzymes β-carotene oxygenase 2 (BCO2), involved in the catalytic activities of both provitamin and non-provitamin A carotenoids which results in the formation of apo-carotenoids [28]. Hence the mitochondria are likely to be an important target for cellular protection by carotenoids. BCO2 has a role to play in mitochondrial oxidative stress, low grade inflammation and metabolic dysfunction [29]. Deficiency of BCO2 leads to the overproduction of superoxide in the mitochondria, suppresses mitochondrial superoxide dismutase levels and impairs mitochondrial respiratory complexes [29]. Other studies have shown that BCO2 is essential in metabolic homeostasis and mitochondrial respiration in mammals [28]. This data indicates that BCO2-driven apo-carotenoids may serve as a substrate for the synthesis of compounds known for their antioxidant activities. The rationale behind mitochondrial localisation of BCO2 is likely to be multifaceted and involve complex interactions between carotenoid metabolism, mitochondrial function, and cellular signalling pathways. It is also possible that the presence of BCO2 in mitochondria facilitates localised regulation of carotenoid metabolism, preventing the release of certain metabolites into the cytoplasm. Provitamin A carotenoids and apocarotenoids (produced by the oxidative cleavage of carotenoids) by cytosolic enzyme BCO1 are converted into retinal (Figure 2). Mutations in enzymes, BCO1 and BCO2 are linked to inflammation and metabolic disorders including type 2 diabetes, obesity, and non-alcoholic fatty liver [30,31].

Higher plasma levels of carotenoids are associated with decreased risk of chronic diseases such as obesity, diabetes, and some types of cancer and may contribute to mucosal and gut barrier health. Carotenoids also activate the body's own antioxidant defence mechanism relating to the expression of antioxidant enzymes (glutathione-peroxidase, superoxide dismutase and catalases) [32]. The interaction of carotenoids with transcription factors, such as Nrf-2 is also an important contributing factor [32]. It has been suggested that the protective role of carotenoids could be achieved through activation of vitagenes (genes involved in preserving cellular homeostasis) [33]. The depletion of the macular carotenoids’ lutein, zeaxanthin, and meso-zeaxanthin in the ageing retina has been implicated in age-related macular degeneration (AMD) disease progression [34].

In conjunction with their major antioxidant role, appropriate concentrations of carotenoids provide a variety of biological advantages, including neuroprotective, anti-inflammatory, immunity-boosting effects, and modulation of signalling pathways [35]. Carotenoids such as lutein, zeaxanthin, and meso-zeaxanthin are mainly found in the macula and have shown to be associated with reduced risk of cataract development and AMD [34]. Despite the antioxidant effect of carotenoids, a pro-oxidant role in tumorigenic models has been linked to their anticancer effects. However, a recent meta-analysis study concluded that β-carotene supplementation has no beneficial or harmful effect on cancer incidence [36]. Carotenoids have potential to improve general dental health where local or systemic use of lycopene has shown improvements in periodontal health [37,38]. A protective role of carotenoids against kidney disease has been linked to its ability to reduce oxidative stress and inflammation, which are key factors in the development and progression of renal disorders. Carotenoids are also associated with a reduced risk of obesity due to their potential role in regulating metabolism and reducing inflammation [39]. Data from the 2007–2008 National Health and Nutrition Examination Survey (NHANES) showed lower alpha carotene was associated with greater difficulty maintaining sleep patterns [40]. In addition, previous studies have identified diurnal fluctuations in mitochondrial calcium dynamics that contribute to mROS [41]. This evidence suggests that maintaining mitochondrial health via carotenoids may have potential benefit in sleep.

## Carotenoids and mitochondrial regulation in ageing and age-related diseases

The evolutionary nature of the mitochondrion in maintaining cellular health has rendered it a key organelle in promoting healthy ageing, extending beyond energy generation but also impacting cell fate and function. Oxidative stress, lipid toxicity, deregulated mitophagy, metabolic disturbances and DNA damage are factors involved in ageing-associated mitochondrial dysfunction. It is therefore important to understand the role of mitochondrial homeostasis on the health span and lifespan.

Studies have suggested that carotenoids could be involved in maintaining mitochondrial function and integrity. Lycopene has been shown to be capable of restoring mitochondrial respiratory activity post-Aβ42 insult in rats, thereby reducing mitochondrial oxidative stress [42]. We have previously shown that lutein and zeaxanthin, as well as lycopene, are found in plasma at lower concentrations in AD patients with related vascular comorbidities [26]. Furthermore, AD patients were shown to have higher levels of oxidised phospholipid compared with age-matched controls which was correlated with cognitive function. When treated with carotenoids for 6 months, the level of oxidised phospholipid in sera did not change in AD patients but did increase in control patients [43]. It was hypothesised that oxidative stress, as evidenced by lower concentrations of carotenoids and higher concentrations of oxidised lipids, promoted AD pathology. We have previously confirmed that non-toxic concentrations of oxidised lipid (POVPC — 1 palmitoyl 2 (5′ oxo valeroyl) sn-glycero-3-phosphocholine) impaired mitochondrial metabolism [43,44]. Whether lipid peroxidation in mitochondria is a potential target in the ageing process merits further study.

## Astaxanthin a novel mitochondria regulator

Astaxanthin ((3,3′-dihydroxy-β,β′-carotene-4,4′-dione), a bright-orange to red carotenoid) has been suggested to be the best radical-scavenging antioxidant among mitochondrion-permeable antioxidants [45]. Astaxanthin is found in marine animals such as shrimps, algae, lobster, crab, salmon, and some other organisms [46]. In addition to antioxidant properties, astaxanthin is a potent anti-inflammatory, anti-apoptotic and anti-proliferative compound with ability to lower plasma LDL and increase HDL levels [47]. Astaxanthin contributes to the enhancement and maintenance of mitochondrial activity, improves mitochondrial biogenesis, inhibits mitochondrial fission/fragmentation by activating antioxidant and anti-inflammatory pathways as well as reducing the expression of Drp1 protein [46,47]. By activating the mammalian target of rapamycin pathway, astaxanthin increases mitochondria fusion and reduces fission [48]. Astaxanthin activates leukocytes, providing protection against infection, combat DNA oxidative-damage/induction of apoptosis and activates immune surveillance [48]. The efficacy of astaxanthin has been reported in renal-, hepato-, skin-, eye-related and neurodegenerative diseases, as well as gastrointestinal disorders in animal models [47]. For example, when administered alongside tocopherol locally to streptozotocin-induced diabetic mice (db/db mice), the fasting blood glucose was reduced [49].

Although animal studies, particularly with rodents, provide valuable insights into mechanisms and initial understanding, their limitations necessitate complementing them with human studies. Drawing meaningful conclusions about carotenoids, including astaxanthin, in human tissues requires cautious extrapolation from animal findings. Researchers must be aware of these inherent limitations to enhance the relevance and applicability of carotenoid research to human health. Astaxanthin, a carotenoid with significant varied concentrations in the blood and tissues, exhibits absorption influenced by various factors such as diet, lifestyle, race, dosage, genetics, and co-ingestion with nutrients, especially fats [24,46,50]. Despite these influences, as compared with other carotenoids, astaxanthin's bioavailability is noticeably low in relation to its dosage [51]. Most studies did not detect astaxanthin at the baseline and only after dietary interventions, astaxanthin reached detectable levels between 39 and 52 nmol/l [52,53]. At present, the European Food Safety Authority recommends a daily intake deemed acceptable for astaxanthin at 0.034 mg·kg^−1^·day^−1^, equivalent to 2.38 mg·day^−1^ in a person weighing 70 kg [54]. Therefore, human pharmacokinetic studies of astaxanthin-containing formulations are essential.

Astaxanthin experiences rapid inactivation after oral administration primarily due to its susceptibility to oxidation. Being a carotenoid, astaxanthin is sensitive to the oxidative conditions present in the gastrointestinal tract. Exposure to oxygen and other reactive compounds in the digestive system leads to the degradation of astaxanthin molecules, diminishing their bioavailability and effectiveness. Additionally, its absorption is influenced by the presence of dietary fats. The efficiency of absorption can vary based on the composition of the meal consumed with astaxanthin, impacting its overall bioavailability. The combination of oxidative stress in the digestive environment and the challenges associated with its fat-solubility contributes to the rapid inactivation of astaxanthin after oral intake.

The membrane interactions of carotenoids have been linked to their biological activities and the vertical orientation of astaxanthin in membranes has been suggested to be responsible for its high efficiency in removing free radicals from membranes, and more efficiently in the presence of water-soluble antioxidants, such as vitamin C and/or GSH [55]. As most of the important components of the mitochondrial ETC are located within the mitochondria inner membrane of mitochondria, astaxanthin may protect mitochondrial membranes against oxidative damage caused by ROS by inhibiting the accumulation of lipid peroxides resulting from lipid peroxidation reactions. Astaxanthin's anti-inflammatory property is reported to be through inhibition of cyclooxygenase 2 (COX2) enzyme activities [46]. In addition to the regulation of the COX2 signalling pathway, astaxanthin also affects nitric oxide, prostaglandin E2 and C-reactive protein and suppresses the NF-κB-mediated gene expression of pro-inflammatory cytokines, including TNF-α, IL-6, IL-8, iNOS, IL-β, [51]. Based on a clinical study, astaxanthin was more effective when combined with exercise; hence, may be used in combination with exercise therapy and standard therapeutic interventions [46]. More research is still needed to fully understand the mechanisms, interactions, and potential benefits of astaxanthin in managing age-related diseases.

## Challenges and future of mitochondria-targeted carotenoid treatment

Use of carotenoids as therapeutics is challenging due to their stability and bioavailability. Main challenges in the use of carotenoids include the possibility of carotenoids to exhibit pro-oxidant properties under specific conditions, such as ascorbic acid (>1 mM), production of hydroxy radicals and hydrogen peroxide in the presence of Fe(III) [56]. Reasons why antioxidant therapies show negative or ambiguous results in some clinical trials may be attributed to: (a) The fact that oxidative damage may not be the primary nor only cause of the disease. (b) Lack of personalised medicine as patients do not benefit from the same antioxidant treatment equally. (c) Lower efficiency because of oral administration. (d) Some antioxidants have adverse effects that mask ROS-scavenging activities. These obstacles to the clinical applications of carotenoids will benefit from further investigation.

Some work has been done to synthesise structurally similar stable carotenoid compounds that are recognised by cellular enzymatic pathways [57] and complex formation to improve the solubility of preparations, to deliver carotenoids, and to decrease their toxicity [58]. Application of supramolecular complexes based on water-soluble nanoparticles (NP) is an effective strategy to improve their stability during storage and to enhance bioavailability by modulating their kinetic release from the delivery system [59,60].

Recent development of nanoencapsulation and nano-drug delivery systems opened new opportunities to deliver carotenoids into cells [61,62]. The development of specifically designed NP improved transportation of drugs to the brain especially allowing passing through blood brain barrier [63]. NP-based delivery platforms demonstrated additional advantages such as enhanced carotenoid solubility, reducing dose and enabling brain targeting through intranasal route. Astaxanthin could be targeted and delivered to inflamed cells or areas to achieve a high concentration after endocytosis of small doses by loading into tailored nanoparticles to protect their structure during storage or transport [64].

One drawback in these general delivery systems is the bioavailability within mitochondria. Owing to critical steps in carotenoid metabolism that take place at the inner mitochondrial membrane [65]. Mitochondria targeted carotenoid delivery should provide more protection to cells as it has been suggested that compounds with singlet oxygen — scavenging properties should be directed to target tissue or subcellular sites for efficient removal of ROS without disrupting or eliminating essential redox signalling molecules.

Targeted transport into mitochondria using different delivery systems such as lipophilic cations, liposomes, or peptides have been investigated for wide range of drugs including antioxidants [66]. However, liposomal encapsulation efficiency and *in vitro* release of carotenoids is reported to be varied depending on their structural characteristics [67,68]. For example, lutein doped with cationic liposomes showed better *in vitro* release stability compared with β-carotene [68].

While mitochondria-targeted delivery of carotenoids may exert benefits to overcome oxidative stress observed in many diseases, careful administration is needed to control mitochondrial activity. Carotenoids that are delivered into cells are transported to mitochondria with the aid of lipid transporting proteins. ASTER-B also known as GRAMD1B (GRAM Domain-Containing 1B) contains a steroidogenic acute regulatory protein-related lipid transfer (StART) domain that is responsible for transporting lipids to endoplasmic reticulum and mitochondria [69]. Recent work suggested that ASTER-B preferentially binds oxygenated carotenoids such as zeaxanthin and facilitates transport to mitochondria via non-vesicular route [70]. ASTER-B channel transports hydroxylated carotenoids such as zeaxanthin and lutein into mitochondria to be metabolised by BCO2 [69,71].

## Conclusions

Targeting mitochondrial metabolism as a potential therapeutic approach or as a redox solution in oxidative damage related diseases is promising for healthy ageing as mitochondria are the main sites of ROS production as well as the primary targets of ROS. Exercise and diet, including carotenoids regulate ROS and inhibit mitochondrial fission whilst promoting mitochondria fusion, biogenesis and improve mitochondria function. It is important to understand the mechanisms by which carotenoids improve mitochondrial integrity and function, as well as to investigate the optimal amounts, combinations, and delivery for mitigating ROS related diseases.

## Perspectives

- ROS increases mitochondrial dysfunction by compromising the antioxidant status and increasing mitochondria fission.
- Targeting mitochondrial metabolism is promising in the treatment of age-related diseases.
- Better understanding of the optimal dose, mechanism of action and delivery of carotenoids would be useful in ameliorating ROS-dependent mitochondrial dysfunction.

## Open Access

Open access for this article was enabled by the participation of Aston University in an all-inclusive *Read & Publish* agreement with Portland Press and the Biochemical Society under a transformative agreement with JISC.

## Abbreviations

AMD: age-related macular degeneration
AMPK: AMP-activated kinase
AD: Alzheimer's disease
ATP: adenosine triphosphate
BCO2: β-carotene oxygenase 2
COX2: cyclooxygenase 2
Drp1: dynamin-related protein 1
ETC: electron transport chain
HDL: high-density lipoproteins
LDL: low-density lipoproteins
MQC: mitochondrial quality control
mROS: mitochondrial ROS ()
NF-κB: nuclear factor kappa-light-chain-enhancer of activated B cells
Nrf2: nuclear factor erythroid 2-related factor 2
ROS: reactive oxygen species
TNF: tumour necrosis factor
